# Correlation of Gut Microbiome Between ASD Children and Mothers and Potential Biomarkers for Risk Assessment

**DOI:** 10.1016/j.gpb.2019.01.002

**Published:** 2019-04-23

**Authors:** Ning Li, Junjie Yang, Jiaming Zhang, Cheng Liang, Ying Wang, Bin Chen, Changying Zhao, Jingwen Wang, Guangye Zhang, Dongmei Zhao, Yi Liu, Lehai Zhang, Jun Yang, Guimei Li, Zhongtao Gai, Lei Zhang, Guoping Zhao

**Affiliations:** 1 Shandong Children’s Microbiome Center, Qilu Children's Hospital of Shandong University, Jinan 250022, China; 2 Department of Pediatrics, Shandong Provincial Hospital Affiliated to Shandong University, Jinan 250021, China; 3 Beijing Advanced Innovation Center for Big Data-Based Precision Medicine, Beihang University, Beijing 100191, China; 4 Research Institute of Pediatrics, Qilu Children’s Hospital of Shandong University, Jinan 250022, China; 5 Institute of Child Health Care, Qilu Children’s Hospital of Shandong University, Jinan 250022, China; 6 College of Life Science, Qilu Normal University, Jinan 250200, China; 7 School of Information Science and Engineering, Shandong Normal University, Jinan 250358, China; 8 School of Chemistry, Beihang University, Beijing 100191, China; 9 CAS Key Laboratory of Synthetic Biology, Shanghai Institutes for Biological Sciences, Chinese Academy of Sciences, Shanghai 200031, China

**Keywords:** Autism spectrum disorders, Gut microbiome, Biomarker, Mother–child pair, Microbiota-gut-immune-brain axis

## Abstract

Variation of maternal gut microbiota may increase the risk of **autism spectrum disorders** (ASDs) in offspring. Animal studies have indicated that maternal gut microbiota is related to neurodevelopmental abnormalities in mouse offspring, while it is unclear whether there is a correlation between gut microbiota of ASD children and their mothers. We examined the relationships between **gut microbiome** profiles of ASD children and those of their mothers, and evaluated the clinical discriminatory power of discovered bacterial **biomarkers**. Gut microbiome was profiled and evaluated by 16S ribosomal RNA gene sequencing in stool samples of 59 **mother–child pairs** of ASD children and 30 matched mother–child pairs of healthy children. Significant differences were observed in the gut microbiome composition between ASD and healthy children in our Chinese cohort. Several unique bacterial biomarkers, such as Alcaligenaceae and *Acinetobacter*, were identified. Mothers of ASD children had more Proteobacteria, Alphaproteobacteria, Moraxellaceae, and *Acinetobacter* than mothers of healthy children. There was a clear correlation between gut microbiome profiles of children and their mothers; however, children with ASD still had unique bacterial biomarkers, such as Alcaligenaceae, Enterobacteriaceae, and *Clostridium*. Candidate biomarkers discovered in this study had remarkable discriminatory power. The identified patterns of mother–child gut microbiome profiles may be important for assessing risks during the early stage and planning of personalized treatment and prevention of ASD via microbiota modulation.

## Introduction

Autism spectrum disorders (ASDs) are considered a heterogeneous set of neurobehavioral disorders that are characterized by social deficits, repetitive behaviors, and cognitive inflexibility in early childhood, which present a substantial challenge to diagnosis and treatment [1]. The incidence of ASD is steadily increasing in various countries, putting a heavy burden on individuals, families, and the society [2], [3]. The past decade has seen good progress in identifying genetic risk factors for ASD that point to specific mechanisms and pathways for related behavioral deficits [4], [5]. Genetic studies have revealed that there is a strong genetic influence on the development of autism [6], however, their risk effects are highly variable, and often related to factors besides autism [7]. Although such understanding should be applied to clinical nursing optimization, there is substantial discrepancy between current knowledge and clinical application [8]. It has been recently recognized that genetics alone does not explain the underlying cause in many cases [9], [10]. Although the causes of ASD are not yet known, it is generally believed that genetic, epigenetic, and environmental risk factors interact and all play roles in the development of ASD [11], [12].

Epidemiological and animal-based studies have suggested that inflammation-induced maternal immune activation, prenatal exposure to immune challenges, and maternal obesity, stress, and gastrointestinal symptoms during pregnancy play roles in perinatal neurodevelopmental brain damage and contribute to an increased risk of subsequent neuropsychiatric disorders, such as ASDs [13], [14], [15], [16], [17]. In recent years, there has been a lot of epidemiological and biological evidence that prenatal factors trigger a more active immune state in the mother, which is associated with the development of autism [9], [13], [14], [15], [16], [18], [19], [20]. Multiple animal studies have also indicated that in addition to genetic influences, the maternal gut microbiota may play an essential role in the occurrence of autism in offspring during gestation, and exhibit continuous correlations and long-term pathological consequences during development [9], [16], [21]. These studies have shown that the gut–brain axis may be formed through bidirectional communication among the nervous, endocrine, and immune systems. The imbalance in the composition and quantity of intestinal microorganisms may affect both the intestinal nervous system and the central nervous system, indicating that the microbiome-intestinal-immune-brain axis and maternal intestinal microbiome may be pathogenic risk factors for ASD [9], [18], [19], [20]. Epidemiological studies have further demonstrated that alterations in the composition and metabolic products of the gut microbiome may contribute to ASD pathophysiology, and several recently published studies on autistic rodents caused by prenatal insults in female rats support this view [22]. Intriguingly, altering the gut microbiota and providing gut commensal bacteria (microbial reconstitution) have been shown to reverse maternal factor-induced social and synaptic deficits in offspring and have beneficial effects on ASD behaviors in both mice and humans [9], [16], [21]. Evidence for the link between ASD and abnormalities in gut microbial function has been accumulating; however, epidemiological and animal studies alone may not be sufficient enough to determine the actual correlations and mechanisms in humans [18].

Multiple studies using clinical samples have reported differences in microbiota composition and specific enteric bacteria existing in fecal, ileal, or duodenal samples from ASD children, providing potential diagnostic and therapeutic targets [23], [24], [25], [26], [27], [28], [29], [30], [31], [32], [33], [34]. The majority of these studies focused more on evidence-based correlation analysis of microbiome with ASD than causality questions, using cohorts of mostly Caucasians. Various types of gut microbes have been identified as biomarkers, such as *Clostridium tetani*, *Desulfovibrio* spp., *Bacteroides vulgatus*, Alcaligenaceae, *Sutterella* spp*.*, *Ruminococcus gnavus*, *Ruminococcus torques*, *Lactobacillus* spp., *Desulfovibrio* spp., Fusobacteria, Verrucomicrobia, Eubacteriaceae, Lachnospiraceae, Sutterellaceae, Enterobacteriaceae, *Bifidobacterium*, *Faecalibacterium*, *Prevotella*, *Coprococcus*, unclassified Veillonellaceae, *Burkholderia*, *Neisseria*, *Alistipes*, *Bilophila*, *Dialister*, *Parabacteroides*, *Veillonella*, *Collinsella*, *Corynebacterium*, and *Dorea*. A recent study has investigated the differences between fecal microbial communities of 35 Chinese ASD children and 6 typical development (TD) children. No difference in alpha diversity was found between the two groups, while relative abundance of *Sutterella*, *Odoribacter*, and *Butyricimonas* in the ASD group was much higher. In contrast, abundance of *Veillonella* and *Streptococcus* was significantly reduced compared to that of the TD group [35]. However, to the best of our knowledge, no study has investigated the gut microbiome profiles of mother–child pairs of ASD children and evaluated their correlations at the same time. Thus, it is still unclear how the gut microbiome varies between mothers of ASD children and those of healthy children and whether maternal gut bacterial communities are associated with the gut microbiome profiles of ASD children. Additionally, the unique features of the gut microbiome of ASD children in comparison with their mothers or healthy children have not yet been identified. Therefore, in this study, we examined the relationships among the gut microbiome profiles of ASD children and their mothers and evaluated the potential clinical discriminatory power of the discovered bacterial biomarkers. This pilot study suggests the role of gut microbiota in autism and could serve as a basis for further investigation of the combined effect of genetic, microbial, and hormonal changes for development and clinical manifestation of autism.

## Results

### Study population

We enrolled 59 ASD children (ASD-Cs) and their mothers (ASD-Ms), together with 30 matched healthy (neurotypical) children (H-Cs) and their mothers (H-Ms), for the current gut microbiome study. The average age of ASD-Cs and H-Cs at the time of sample collection was 4 (range, 2–7) and 5 (range, 2–10) years old, respectively. The average age of ASD-Ms and H-Ms at the time of sample collection was 33 (range, 26–38) and 31 (range, 27–42) years old, respectively. The characteristics of the children and mothers are reported in Table 1 and Table S1. In the subsequent results and analyses, we defined the groups as ASD-C, ASD-M, H-C, H-M, ASD-M+C (for mother–child pairs of ASD children), and H-M+C (for mother–child pairs of healthy children).

**Table 1:** **Characteristics of study participants**

**Parameter**	**ASD-C**	**H-C**	**ASD-M**	**H-M**
No. of participants	59	30	59	30
Age range (mean), years	2–7 (4)	2–10 (5)	26–38 (33)	27–42 (31)

Gender				
Female	9	10	59	30
Male	50	20	N/A	N/A

History of GI problem [No. (%)]				
Yes	30 (50)	7 (23)	17 (29)	4 (13)
No	29 (50)	23 (77)	42 (71)	26 (87)

*Note*: ASD, autism spectrum disorder; ASD-C, ASD child; H-C, healthy child; ASD-M, mother of ASD child; H-M, mother of healthy child; GI, gastrointestinal.

### ASD children harbored an altered gut microbiome in the Chinese cohort

**Figure 1:**
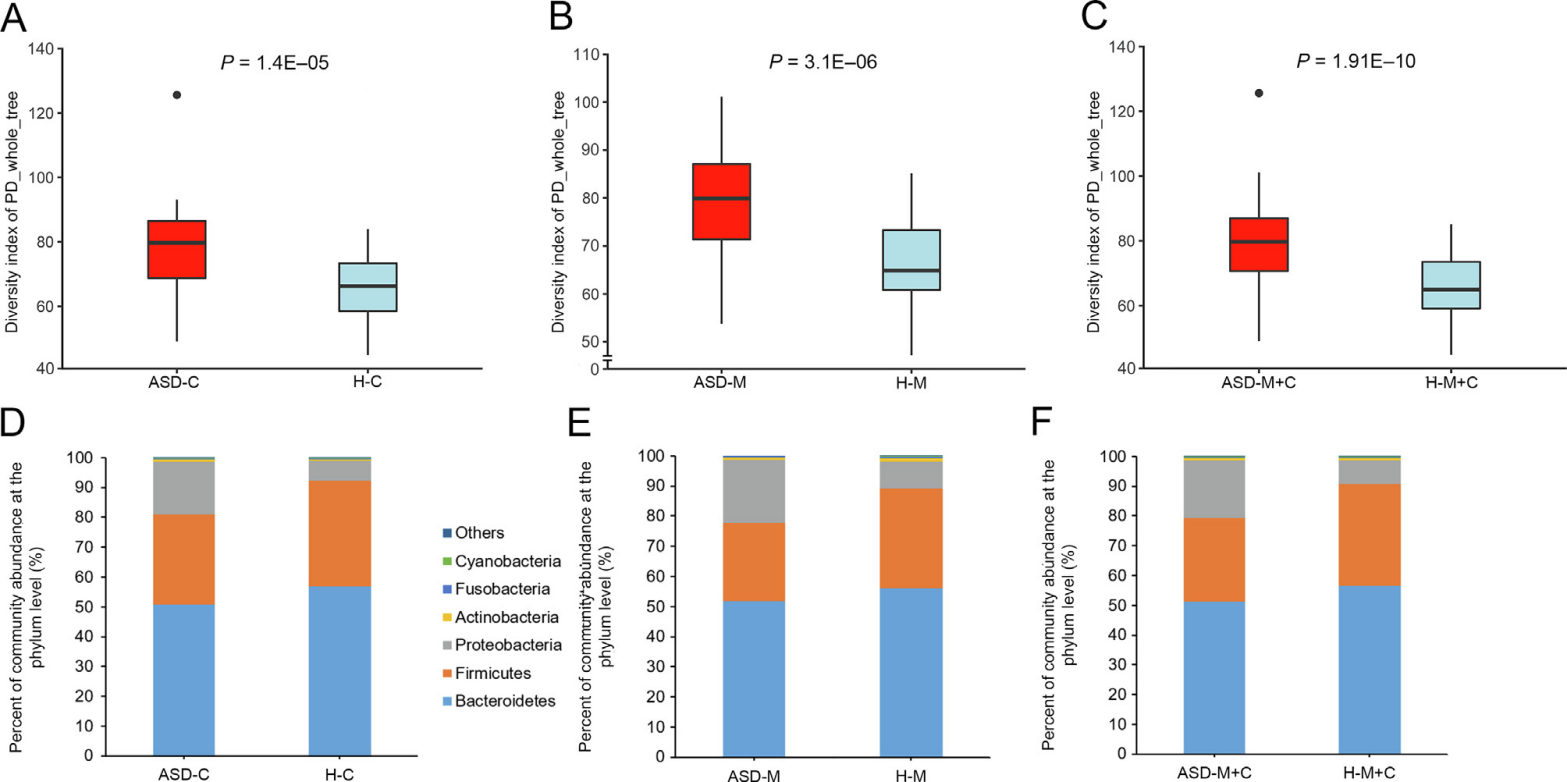
**Comparison of the alpha diversity and relative abundances at the phylum level based on the OTU profile** Comparison of the alpha diversity was evaluated using PD_whole_tree based on the OTU profile between the autism groups and the control groups and shown in the top panels for ASD-C *vs*. H-C (**A**), ASD-M *vs*. H-M (**B**), and ASD-M+C *vs*. H-M+C (**C**). *P* values were calculated using the Wilcoxon rank-sum test. The relative abundances of different taxa at phylum level were shown in the bottom panels for ASD-C *vs*. H-C (**D**), ASD-M *vs*. H-M (**E**), and ASD-M+C *vs*. H-M+C (**F**). OTU, operational taxonomic unit. ASD-C, ASD child; ASD-M, mother of ASD child; H-C, healthy child; H-M, mother of healthy child; ASD-M+C, mother–child pair of ASD child; H-M+C, mother–child pair of healthy child.

**Figure 2:**
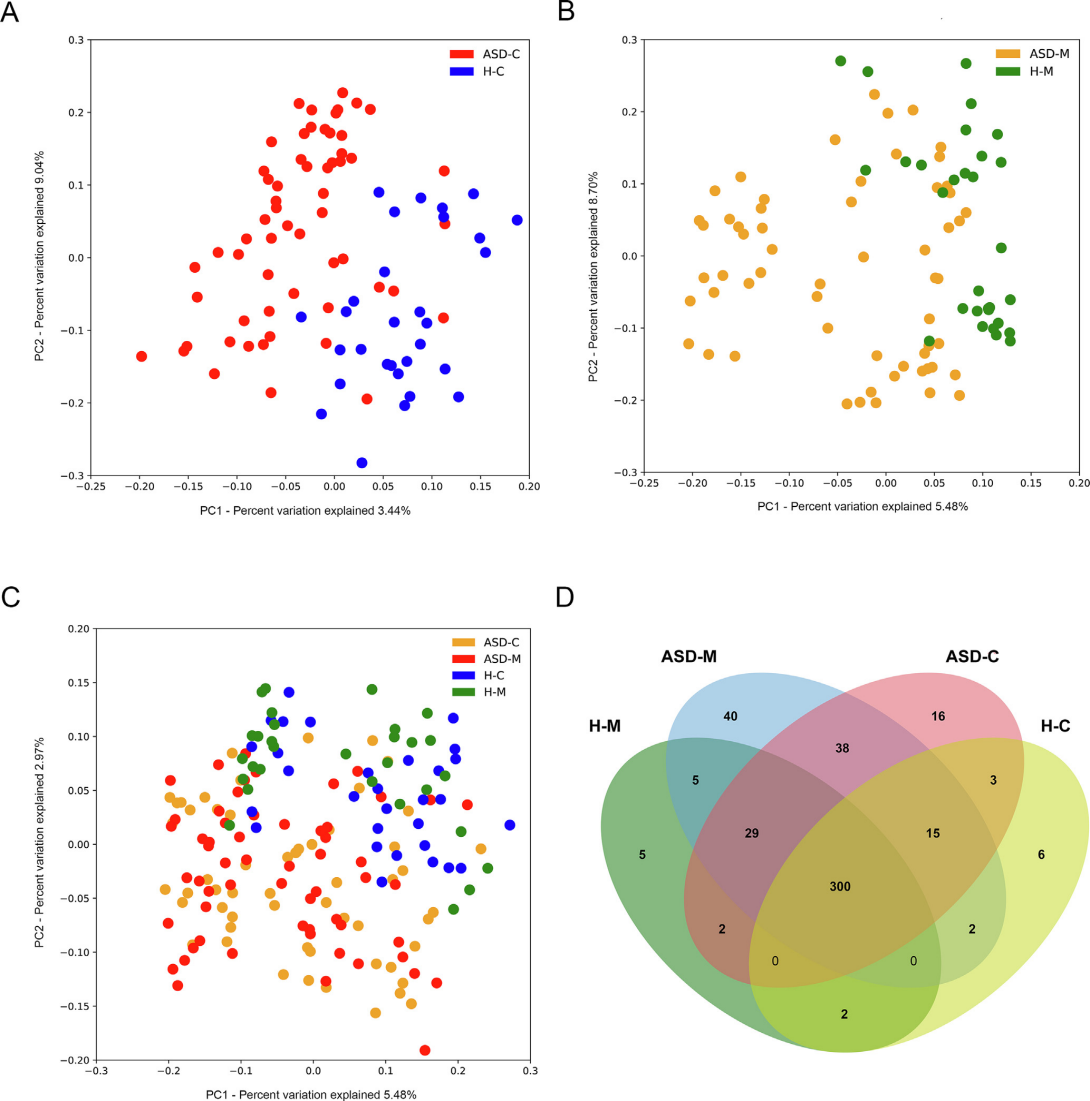
**Microbiome community and Venn diagram analysis** PCoA of bacterial beta diversity based on the unweighted UniFrac distances. ASD-C *vs*. H-C (**A**); ASD-M *vs*. H-M (**B**); ASD-M+C *vs*. H-M+C (**C**). **D.** Venn diagram displaying the degree of overlap of bacterial OTUs among ASD-C, ASD-M, H-C and H-M. PCoA, principal coordinate analysis.

**Supplementary Figure S1:**
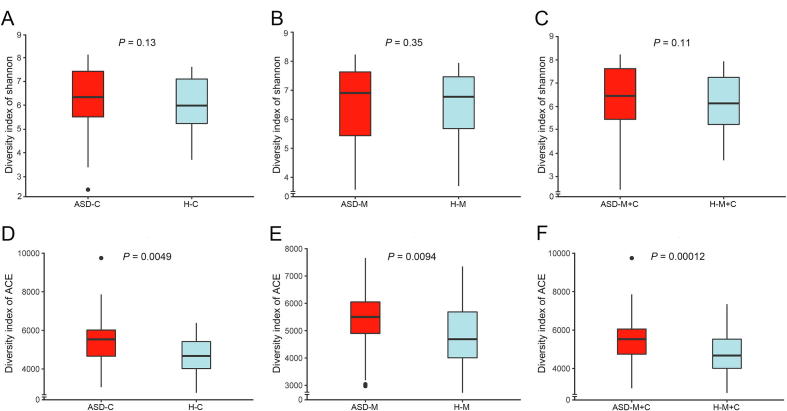
**The diversity and richness of the gut microbiota in the autism groups and the control groups** The Shannon index of the gut microbiome in ASD-C and H-C (**A**), ASD-M and H-M (**B**), as well as ASD-M+C and H-M+C (**C**); and the ACE index of the gut microbiome in ASD-C and H-C (**D**), ASD-M and H-M (**E**), as well as ASD-M+C and H-M+C (**F**). ACE, abundance-based coverage estimator.

**Supplementary Figure S2:**
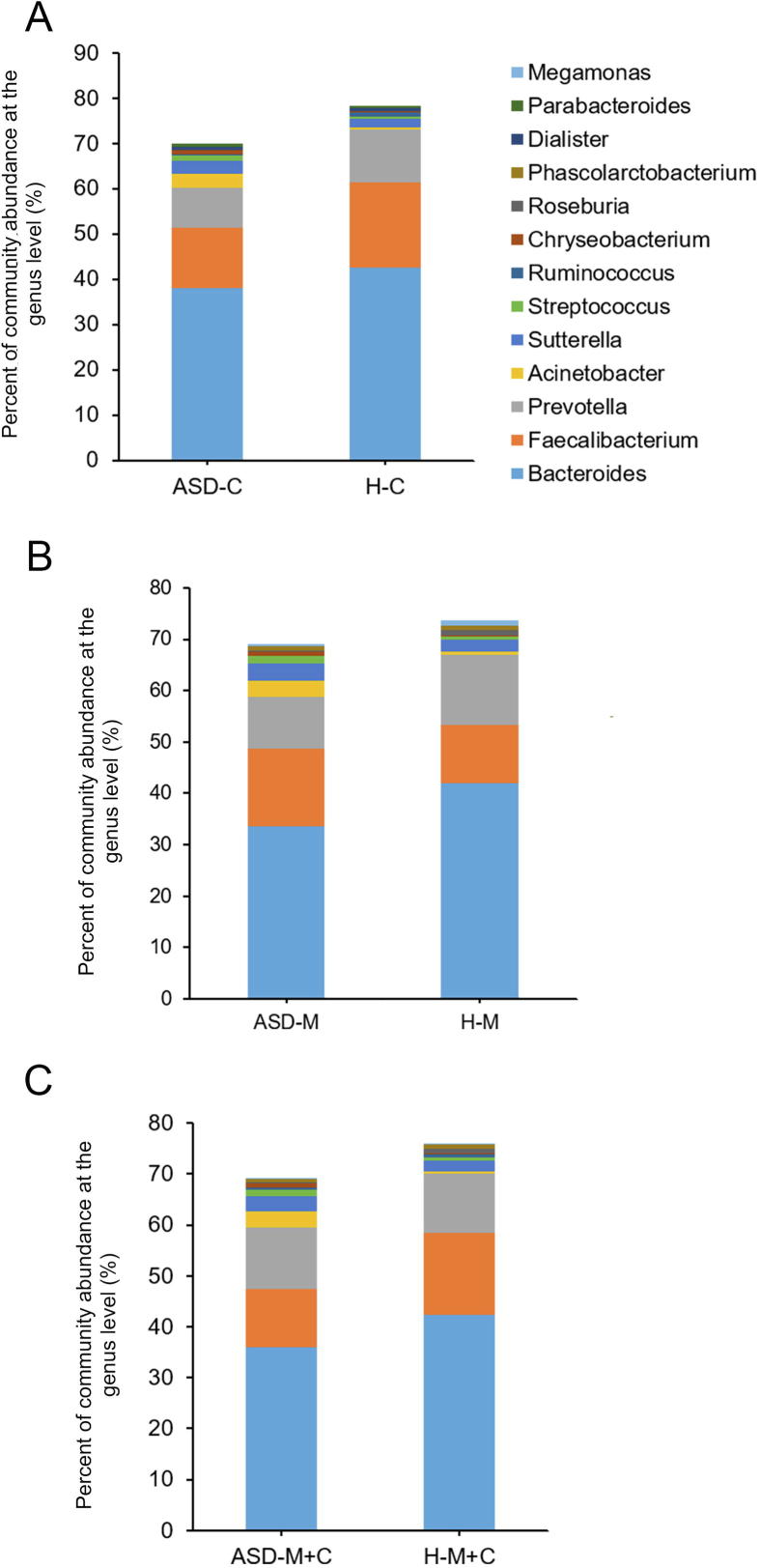
**Comparison of relative taxonomic abundance between the autism groups and the control groups at genus level** Comparison of relative taxonomic abundance between different groups at the genus level (average of each group). **A.** ASD-C *vs.* H-C. **B.** ASD-M *vs.* H-M. **C.** ASD-M+C *vs.* H-M+C.

For characterization of the gut microbiome associated with ASD, we compared the alpha diversity between the ASD-C and H-C groups. We found significant increases in bacterial richness (*P* < 0.01, Figure 1A and Figure S1D) in ASD-Cs and nonsignificant difference in bacterial diversity between two groups (*P* = 0.13, Figure S1A). To further explore the characteristics of intestinal bacterial community of ASD-Cs, we assessed the relative taxon abundance in the microbiota of the ASD-C and H-C groups. The total distribution of bacterial taxonomy showed no significant variations in the bacterial communities between ASD-Cs and H-Cs at the phylum level, as characterized by a similar Firmicutes/Bacteroidetes ratio (*P* > 0.05, Figure 1D); however, a significant increase in the relative abundance of Proteobacteria was observed in the ASD-C group compared with the H-C group (*P* < 0.01). We also compared differences in the taxa at the genus level (Figure S2A). Analysis of the beta diversity based on the unweighted UniFrac distances showed that the microbiome of the ASD-C group was distinct from that of the H-C group. We further performed an analysis of similarities (ANOSIM), and the results indicated that the structure of the gut microbiome of the ASD-C group was significantly different from that of the H-C group (ANOSIM, *r* = 0.197, *P* < 0.01, unweighted UniFrac, Figure 2A).

**Figure 3:**
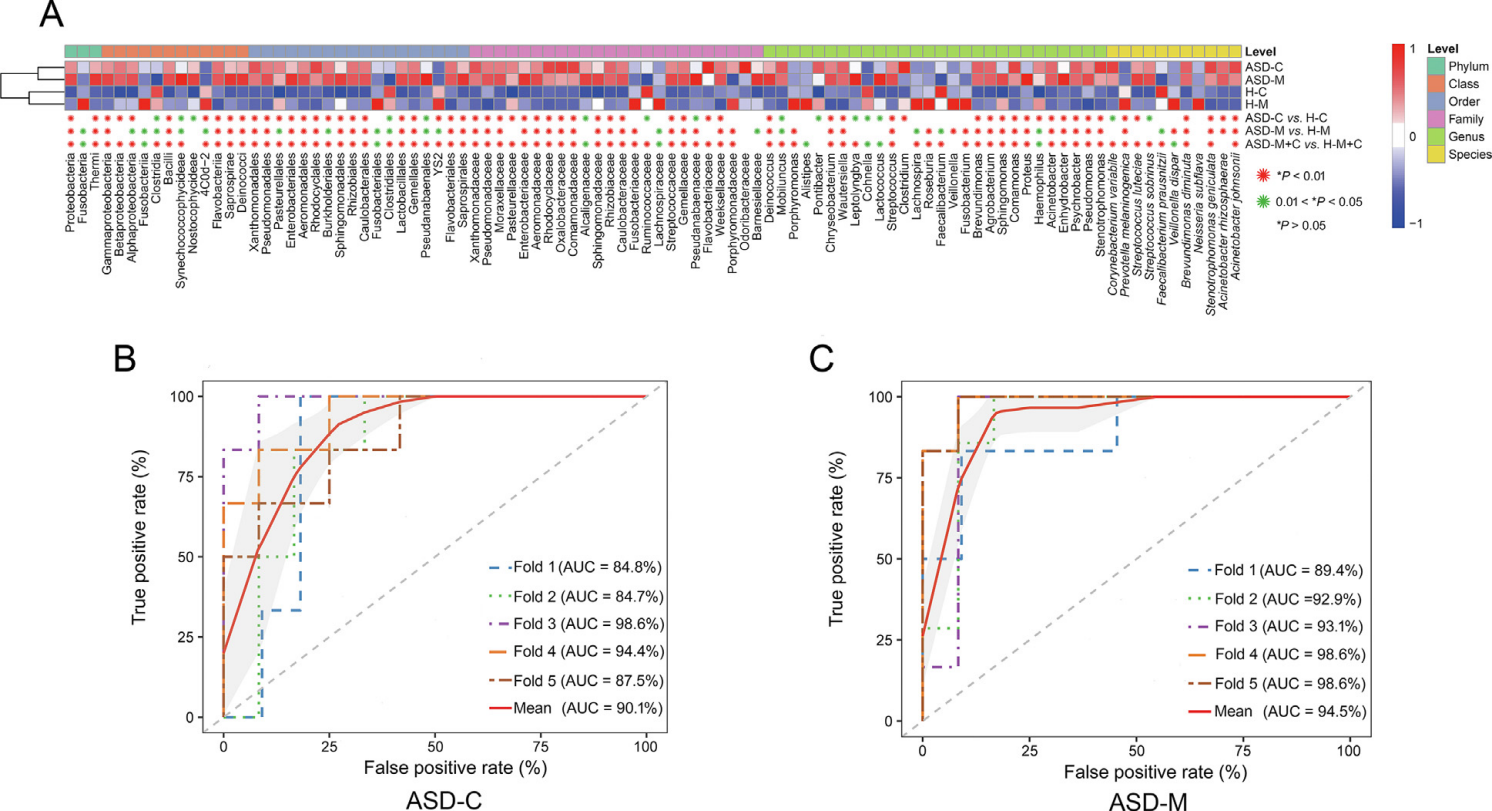
**The relative abundance of the OTUs and ROC curves** **A.** The relative abundance of the top 96 most different OTUs across groups (LDA score >2 and adjusted *P* < 0.1) according to the Wilcoxon rank sum test. The abundance profiles are transformed into Z scores by subtracting the average abundance and dividing the standard deviation of all samples. Z score is negative (shown in blue) when the raw abundance is lower than the mean. OTUs with adjusted *P* < 0.01 and *P* < 0.05 are marked in red and green, respectively. **B.** ROC curve with adjusted *P* < 0.01, (Wilcoxon rank sum test) and LDA score >3 (LEfSe analysis). 5 biomarkers were selected to predict the risk of disease in children with autism. These include Betaproteobacteria, Burkholderiales, Pseudomonadales, Moraxellaceae, and *Acinetobacter*. **C.** ROC curve with adjusted *P* < 0.01 (Wilcoxon rank sum test) and LDA score >3 (LEfSe analysis). 6 biomarkers were selected to predict the risk of disease in children’s mothers. These biomarkers are Flavobacteriia, Gammaproteobacteria, Flavobacteriales, Weeksellaceae, Enterobacteriaceae, and Enterobacteriales. The SVM classifier from R package e1071 was adopted to perform the classification analysis for the selected biomarkers. Five-fold cross-validation was used to evaluate the performance of the predictive model. The ROC curves as well as the AUC value was calculated using the ROCR R package. *P* values were adjusted by FDR. ROC, receiver operating characteristic; LDA, linear discriminant analysis; LEfSe, linear discriminant effect size; AUC, area under the curve; FDR, false discovery rate.

**Supplementary Figure S3:**
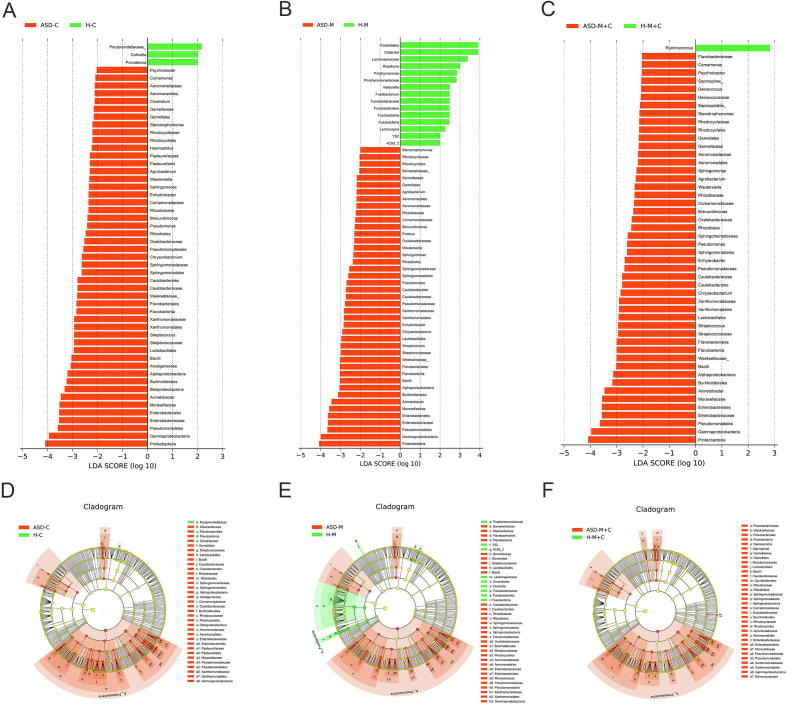
**Comparison of relative taxonomic abundance among ASD-C, H-C, ASD-M, and H-M at the level of phylum (A) and genus (B)**

Linear discriminant effect size (LEfSe) analysis between the ASD-C and H-C groups revealed the signature microbiome profiles and predominant bacterial biomarkers of ASD children. Significant increases in the relative abundance of *Enhydrobacter*, *Chryseobacterium*, *Streptococcus*, and *Acinetobacter* (at the genus level), as well as *Acinetobacter rhizosphaerae* and *Acinetobacter johnsonii* (at the species level), in addition to a significant reduction in *Prevotella melaninogenica* (at the species level), were observed in the ASD-C group in comparison with the H-C group, as indicated by the linear discriminant analysis (LDA) (LDA score >3, Figure 3A). All potential biomarkers (LDA score >2) are shown in Figures S3A and S3D and listed in Table S2.

### Mothers of ASD children harbor an altered gut microbiome

To identify the differences in gut microbiome between mothers of ASD-Cs and H-Cs, we compared the alpha and beta diversities between ASD-Ms and H-Ms. Distinct gut microbiome profiles were revealed. Analysis of the alpha diversity showed a significant increase in bacterial richness of ASD-Ms (*P* < 0.05, Figure 1B and Figure S1E) and nonsignificant difference in bacterial diversity (*P* = 0.35, Figure S1B). We also analyzed the relative abundance of microbes in the gut microbiome between ASD-Ms and H-Ms at the phylum level (Figure 1E) and genus level (Figure S2B). Then, the analysis of beta diversity based on the unweighted UniFrac distances revealed that the microbiome of ASD-Ms was significantly different from that of H-Ms (ANOSIM, *r* = 0.248, *P* < 0.01, unweighted UniFrac, Figure 2B). LEfSe analysis further confirmed these significant differences. Notably, a significant increase in the relative abundances of Moraxellaceae and Enterobacteriaceae (at the family level) and *Acinetobacter* (at the genus level) and a significant reduction in *Faecalibacterium* were observed in the ASD-M group, in comparison with the H-M group (LDA score >3, Figure 3A). All potential biomarkers (LDA score >2) are shown in Figures S3B and S3E and are listed in Table S2.

### ASD children harbored unique bacterial biomarkers

**Figure 4:**
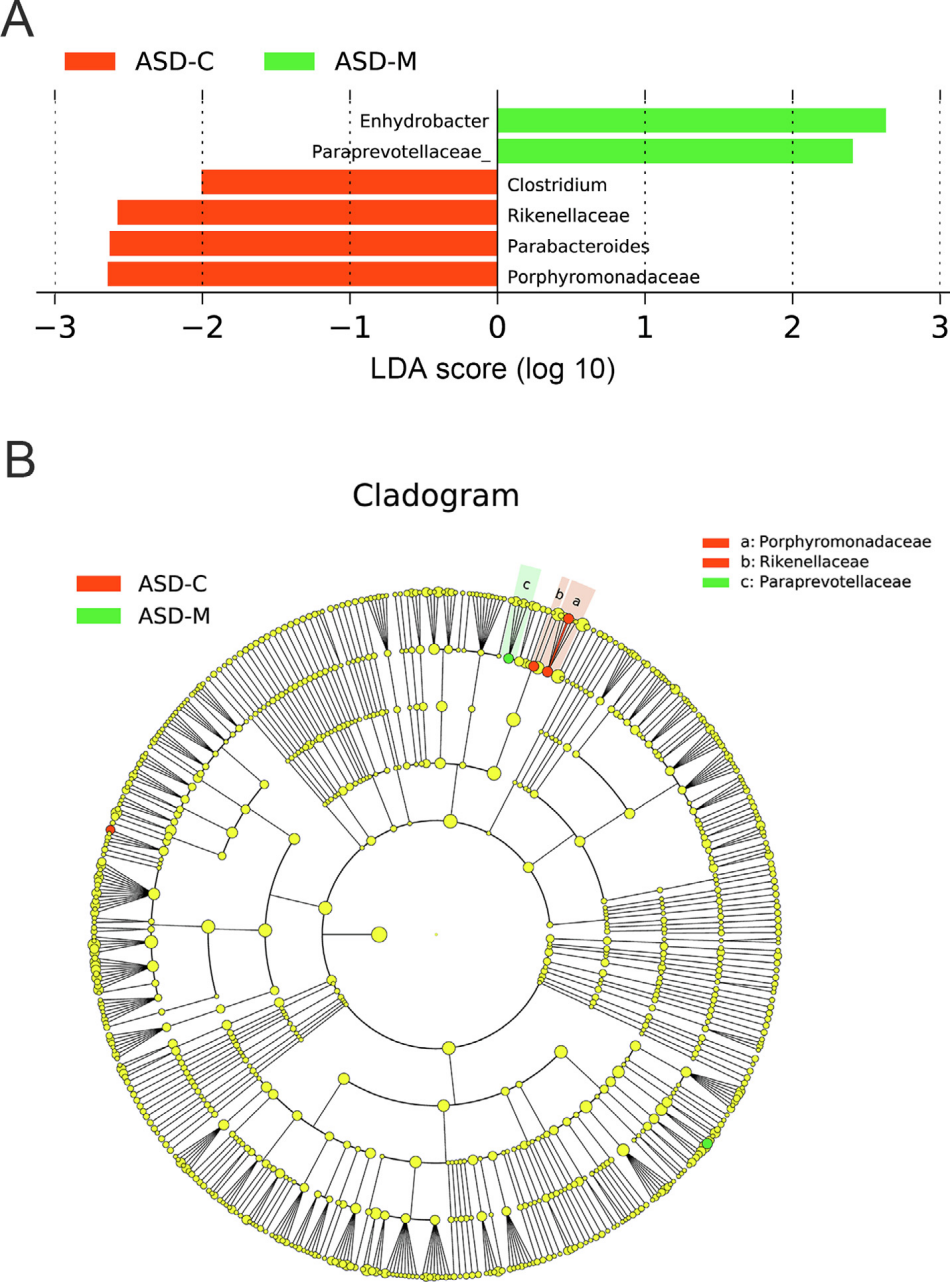
**LEfSe analysis between ASD-C and ASD-M groups** **A.** Histogram of the LDA scores computed for differentially abundant taxa between ASD-C and ASD-M. The LDA score indicates the effect size and ranking of each differentially abundant taxon. **B.** The enriched taxa in ASD-C and ASD-M gut microbiome represented in the cladogram. The central point represents the root of the tree (Bacteria), and each ring represents the next lower taxonomic level (phylum to genus: p, phylum; c, class; o, order; f, family; g, genus). The diameter of each circle represents the relative abundance of the taxon.

For characterization of the gut microbiota between mother–child pairs, we compared the alpha and beta diversities between ASD-M+C and H-M+C groups, and again revealed distinct gut microbiome profiles. Analysis of alpha diversity showed that the sequence-based boxplot based on the PD_whole_tree was nearly asymptotic, and Wilcoxon rank-sum tests demonstrated significant differences in diversity in the ASD-M+C and H-M+C groups (*P* < 0.01, Figure 1C). Abundance-based coverage estimator (ACE) indexes also confirmed these findings (Figure S1F). However, there was no significant difference in Shannon index (Figure S1C). Analysis of beta diversity based on the unweighted UniFrac distances revealed that the microbiome of the ASD-M+C group clustered apart from that of the H-M+C group (ANOSIM, *r* = 0.191, *P* < 0.01, Figure 2C). We then used the identified significant bacterial biomarkers to evaluate the correlations and revealed distinct clustering of children and mothers’ gut microbiomes in the two groups (Figure 3A, Figures S3C and S3F). We further analyzed the different gut microbiome structures between ASD children and their mothers and found that relative abundance of genus *Clostridium* was increased in ASD children (Figure 4).

Furthermore, the number and identity of the shared operational taxonomic units (OTUs) were evaluated through Venn diagrams. As shown in Figure 2D and Table S3, the similarity of the gut microbiome in the ASD-M+C group was higher than that in the H-M+C group. *Bacteroides ovatus* and *Abiotrophia* were found in the H-M+C group. *Epulopiscium*, *Sphingobium xenophagum*, *Anaeroplasma*, *Adlercreutzia*, Solirubrobacterales, *Mesorhizobium*, *Hydrogenophilus*, *Salinicoccus*, and Promicromonosporaceae were only found in ASD children.

### The discovered bacterial biomarkers may have potential value in risk assessment

The relative abundance of the top 96 most different OTUs across groups was determined using the criteria of LDA score >2 and adjusted *P* < 0.1 by Wilcoxon rank sum test (Figure 3A). The signature OTUs across groups at different *P* value stringency are marked in Figure 3A, and the number of these OTU signatures are summarized in Table S3. According to stringent criteria for adjusted *P* value (*P* < 0.01, Wilcoxon rank sum test) and LEfSe analysis (LDA score >3), candidate biomarkers were selected to predict the risk of disease in children with autism; candidate biomarkers were also selected to distinguish between mothers of ASD children and healthy children. Five biomarkers were selected for ASD-C *vs.* H-C, including Betaproteobacteria, Burkholderiales, Pseudomonadales, Moraxellaceae, and *Acinetobacter*; six biomarkers were selected for ASD-M *vs.* H-M, including Flavobacteriia, Gammaproteobacteria, Flavobacteriales, Weeksellaceae, Enterobacteriaceae, and Enterobacteriales.

To explore the potential value of the identified bacterial biomarkers for two levels of clinical discrimination (ASD-C *vs.* H-C, and ASD-M *vs.* H-M), we constructed receiver operating characteristic (ROC) curves and computed the area under the curve (AUC) values. For ASD-C *vs.* H-C, the highest AUC value was 0.944 (4-fold, 95% confidence interval [CI]: 84%–100%) with 83.33% sensitivity and 91.67% specificity (Figure 3B). For ASD-M *vs.* H-M, the highest AUC value was 0.986 (4-fold and 5-fold, 95% CI: 94%–100%), with 100% sensitivity and 100% specificity (Figure 3C). The evaluation of all biomarker combinations is summarized in Table S4. Additionally, we evaluated the effect of age, sex, and history of gastrointestinal (GI) problem on the five candidate biomarkers for ASD-C *vs.* H-C and the effect of age and history of GI problems on the six candidate biomarkers for ASD-M *vs.* H-M. None of these factors had significant effects on the selected candidate biomarkers (Tables S5 and S6).

### KEGG pathways were distinct between gut microbiome of children, mothers, and mother–child pairs

**Figure 5:**
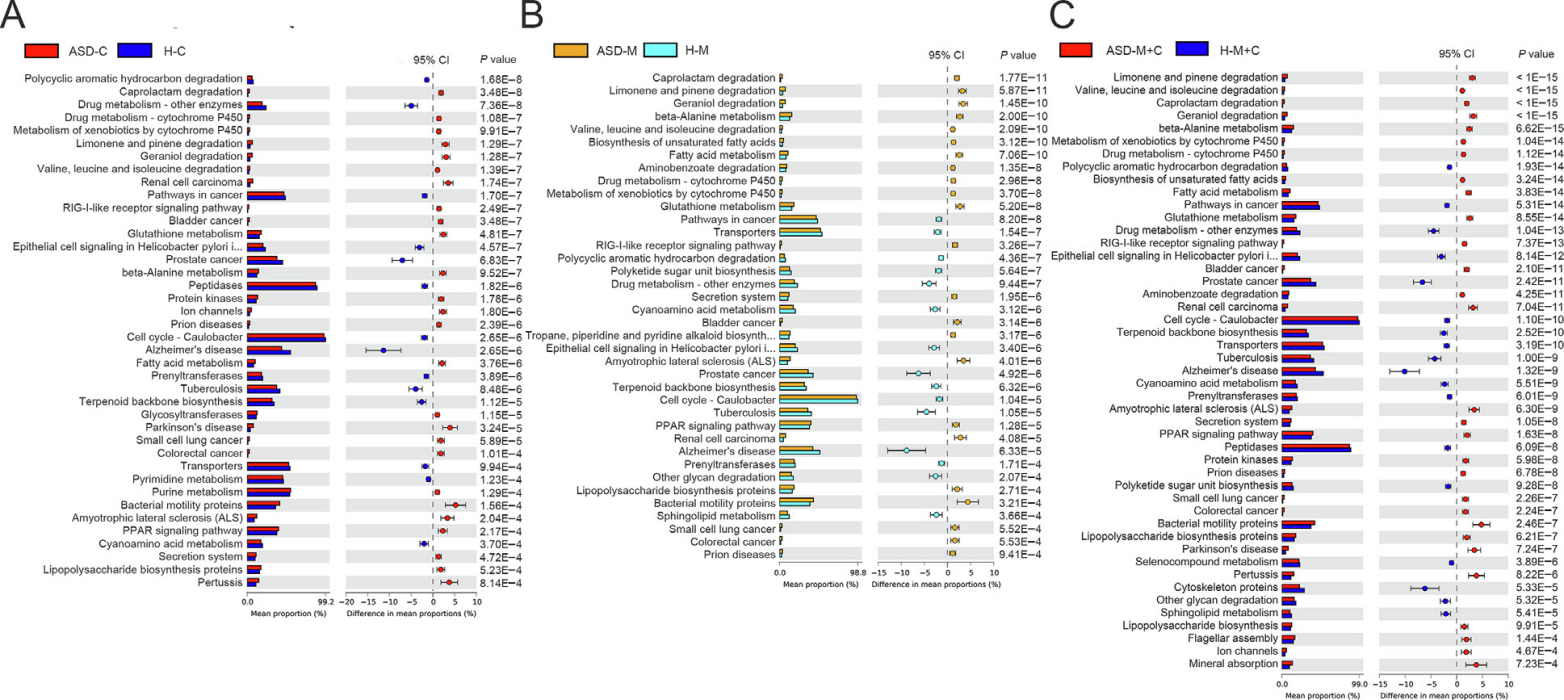
**Predicted metagenome function based on KEGG pathways analysis** Extended error bar plot showed the significantly different KEGG pathways between ASD-C and H-C (**A**), between ASD-M and H-M (**B**), between ASD-M+C and H-M+C (**C**).

The phylogenetic investigation of communities by reconstruction of unobserved states (PICRUSt) method was used to predict the KEGG pathways between the microbiome of ASD patients and healthy subjects, and 40 different KEGG pathways were found. The ASD-C group showed increased activities in some disease pathways, such as pertussis, amyotrophic lateral sclerosis, and Parkinson’s disease, and in bacterial motility proteins. There were 38 different KEGG pathways identified in the ASD-M and H-M groups. The ASD-M group showed increased enrichment in pathways in amyotrophic lateral sclerosis, bacterial motility proteins, renal cell carcinoma, and geraniol degradation (Figure 5).

## Discussion

Many recent studies have confirmed that the causes of autism include genetic and environmental factors [13], [14], [15], [16]. However, research into the environmental factors associated with the development of autism and the molecular mechanisms by which these factors operate is just beginning. Our current study is a pilot exploration to examine the gut bacterial diversity of ASD children using a Chinese cohort, to examine the gut bacterial diversity of the mothers of ASD children, and most importantly, to discover correlations between the microbiome profiles of ASD children and their mothers. We found that the gut microbiota of Chinese ASD children showed significant change, including increased bacterial richness, and different microbiota structures, compared with healthy children. In addition, there were close correlations between the microbiome profiles of mother–child pairs, and ASD children exhibited unique bacterial biomarkers. Finally, the identified bacterial biomarkers exhibited remarkable discriminatory power for differentiating ASD children from neurotypical control children, as well as their mothers.

### ASD children harbored an altered gut microbiome

Consistent with previous clinical studies on the gut microbiome of ASD children, a comparison of the gut bacterial community structures between ASD children and healthy controls in our study revealed significantly shifted microbiome profiles in the feces of ASD children. Further, it identified a set of bacterial biomarkers that varied significantly between the two groups. Previous studies have suggested that the gut microbiome of autistic children may contain harmful genera or species that contribute to the severity of autism symptoms (Tables S7 and S8). A comparison of our results with those of previous studies using other ethnic cohorts found similarities and differences, indicating that the gut microbiome profile and key bacterial signatures may have potential commensal causative microbes and ethnicity-specific characteristics. These potential commensal causative microbes identified in our study and previous studies belong to different taxa. A detailed comparison of the similarities and differences in biomarkers can be found in the Supplemental Material. We have compared our results with one of the most recently published articles on the gut microbiome of Chinese ASD children [35], which examined the differences in fecal microbiota in 35 children with ASD and 6 TD children. In addition to their findings, our study moved one step forward to find correlations between the gut microbiome of ASD children and their mothers. Notably, *Clostridium* and *Streptococcus* were found to be increased in ASD children in our study and most previous studies, in which the potential functions of these two genera have been extensively discussed [23], [28], [36], [37], [38]. *Clostridium*, *Streptococcus*, *Chryseobacterium*, *Haemophilus*, and *Comamonas* are involved in GI disorders, maternal inflammation, maternofetal immune activation, neonatal sepsis, bacteremia or meningitis, acute appendicitis, and childhood vaccination [38], [39], [40], [41], [42]. Williams et al. demonstrated the presence of members of the family Alcaligenaceae in some autism children, but there were no Alcaligenaceae sequences detected in the microbiota from healthy children [29]. Our study also found a significant increase in the abundance of Alcaligenaceae in the gut microbiota from the ASD-C group compared with that in the H-C group. Interestingly, we discovered an evidential increase in *Parabacteroides johnsonii*, a Gram-negative and obligate anaerobe, in the feces from autism children. The genus *Parabacteroides* has been reported to have increased abundance in the feces of ASD children [32]. However, its connection with the pathogenesis of autism remains to be further investigated.

The distinct gut bacterial biomarkers of Chinese ASD children also included four species showing increased abundance, namely, *A. johnsonii*, *A. rhizosphaerae*, *Brevundimonas diminuta*, and *Stenotrophomonas geniculate*. If these unique microbes are found to be causative or consequential factors in Chinese patients with ASD, such findings may facilitate the development of specific diagnostic tests as well as strategies for treatment and prevention of ASD.

### Gut microbiome was different in the mothers of ASD children

As we hypothesized, the gut microbiome varied dramatically between the mothers of ASD children and healthy children. Because the mothers of ASD children were neurotypical, we could only attribute variations in their bacterial biomarkers, *e.g.*, increases in *Streptococcus* and *Acinetobacter* and decreases in *Porphyromonas* and *P. melaninogenica*, to potential GI disorders, maternal inflammation, bacteremia, and antibiotic usage during pregnancy; however, further research is needed to determine the specific causes of these changes. Importantly, we discovered a striking correlation between the microbiomes of mother–child pairs. Previous studies have suggested vertical transmission of the microbiome from the mother to the gut of offspring based on their similarities [43], which could partially explain the similarities in the gut microbiome profiles of the mother–child pairs. In contrast, ASD children harbored unique bacterial biomarkers when compared with their mothers, indicating the potential roles of these microbiota in the etiology of autism. For example, Alcaligenaceae, *Clostridium*, *Haemophilus*, and *Wautersiella* were increased only in the ASD-C group and not in the ASD-M group, whereas Ruminococcaceae and Paraprevotellaceae were decreased only in the ASD-C group and not in the ASD-M group. OTU comparisons also showed that ASD children had unique bacterial biomarkers, such as *Epulopiscium*, *S. xenophagum*, *Anaeroplasma*, *Adlercreutzia*, Solirubrobacterales, *Mesorhizobium*, *Hydrogenophilus*, *Salinicoccus*, *Corynebacterium variabile*, and Promicromonosporaceae.

### Biomarkers may have predictive power on autism

In the current study, Bacteroidetes and Firmicutes were demonstrated to be important phyla. It is worth noting that the vast majority of species in the Bacteroidetes produce propionic acids and other short-chain fatty acids as final products of their metabolism. MacFabe and colleagues have shown clearly when injecting propionic acid or other short-chain fatty acids into rat cerebral ventricles, rats showed unique biological, chemical, and pathological changes, which were the characteristic of autism [44]. Decreasing harmful populations with antibiotics such as vancomycin have been shown to be an important step in improving the symptoms of late onset autism [23], [45].

Significant increases in the abundance of species belonging to Proteobacteria were found in the microbiome of ASD group. Proteobacteria include abundant gram-negative pathogens such as *Escherichia*, *Salmonella*, *Vibrio*, *Helicobacter*, and *Yersinia* [46], which induce inflammatory responses through lipopolysaccharide (LPS) on the cell wall. Previous studies revealed that lipopolysaccharides (LPS) treatment of the schwannoma *in vitro* improved the level of NFκB, IL-1β, pSTAT3, and IL-6 cytokines to activate an immune reaction. Additionally, the connection of Proteobacteria with chronic enteritis was demonstrated based on a mouse model [47].

*Clostridium* and *Streptococcus* were found to be increased in ASD children in our study and most previous studies, in which the potential of these two genera have been extensively discussed [23], [28], [36], [37], [38]. Parracho et al. described the increased abundance of *Clostridium* in the stool from autistic patients based on fluorescent *in situ* hybridization analysis [33]. A possible mechanism of autism pathogenesis is that neurotoxin produced by several *Clostridium* bacteria transits through the vagus nerve into the brain and then blocks neurotransmitter delivery to cause children’s abnormal behavior. In addition, the evident increase in the abundance of pathogenic genera *Wautersiella*, *Agrobacterium*, *Chryseobacterium*, *Streptococcus,* and *Acinetobacter* was found in the gut microbiome of the ASD group. *Wautersiella* has been isolated from various samples, including wound samples, blood samples, respiratory samples from patients with cystic fibrosis, samples from suspected joint prosthesis infection, and the urine of pyelonephritis infants [48]. *Agrobacterium* species are able to infect immunocompromised children to cause bacteremia [49]. *Chryseobacterium* species infect immunocompromised neonates and adults to cause neonatal sepsis, bacteremia, or meningitis, which can be associated with autism pathogenesis [40]. The connection of *Streptococcus* with neurological disorders described in previous studies is consistent with the improved abundance in the ASD group in the current study. *Acinetobacter* spp. leads to serious infections, including sepsis, pneumonia, meningitis, endocarditis, skin infection, and wound infection [50], [51], [52], [53].

We speculated that the decreasing abundance of *Prevotella* [54] and *Ruminococcus* could cause autism pathogenesis through blocking arginine (Arg) metabolism. Argininemia caused by excessive Arg in the blood may lead to neurodegeneration. Further, considering that Arg is one of the substrates of citrulline synthesis, the increase in nitric oxide produced by high levels of Arg-activated citrulline synthesis may inhibit proliferation and differentiation of neural stem cells as neurotoxins [55], [56]. Interestingly, the onset time (the third month to the fourth year) and symptom (the loss of cognitive and athletic competence) of argininemia have striking similarity with autism [57], [58]. In addition, the species of *Prevotella* and *Ruminococcus* were found to be involved in Arg metabolism [59] and had decreased abundance in the microbiome of ASD group in our study. These facts indicated that the suppressed metabolism of Arg caused by reducing *Prevotella* [54] and *Ruminococcus* could lead to high-level nitric oxide, which might cause abnormal neural development and the onset of autism.

### Limitation

Screening for autism carries two major challenges. The first is the need to predict or detect autism in early childhood, even before the onset of symptoms. The second is the ability to differentiate pregnant women at high risk of having autistic children. Based on the discovered biomarkers, ASD children and their mothers could be separated from healthy children and their mothers with high sensitivity and specificity. The discriminatory power of these candidate biomarkers paves the way for establishing fecal microbiome tests for clinical diagnostic and prognostic screening for ASD. However, our study has some limitations. First, the cross-sectional nature of the study prevented us from elucidating the mechanisms and longitudinal view of relevance. Additional large cohort studies are needed to determine the chronological order and to evaluate changes in the gut microbiota of mothers and children. In addition, small sample sizes did not allow subgroup analysis to assess whether the associations of different ASD patients are consistent, as defined by factors such as severity and comorbidities. Separate test and validation cohorts are needed in order to draw the useful conclusions about the discriminatory power of the microbial biomarkers. However, the potential value of these biomarkers for clinical validation and application is not diminished, and nested case–control studies using population-based cohorts are currently under way.

## Conclusions

We found significant differences in the composition of intestinal bacteria between ASD children and healthy children. Although the gut microbiome of ASD children was closely associated with that of their mothers, children with ASD still had unique bacterial biomarkers. Variation of maternal gut microbiota may play a critical role in increasing the risk of ASD in children. The identified similarities and differences in mother–child gut microbiome profiles are important for early assessment of risks and for planning personalized treatment and prevention strategies for ASD via microbiota modulation. Our current study has several noteworthy weaknesses. There was no assessment of diet quality, especially fiber intake effects, on the gut microbiome. The factors of living conditions and history of GI problems were not properly controlled and fully evaluated. Most importantly, a longitudinal study and large cohort validation are warranted to monitor the variation of the gut microbiome of ASD children and their mothers.

## Materials and methods

### Study participants

In this study, which was approved by the Institutional Review Boards of Qilu Children’s Hospital of Shandong University (QCH IRB# 16-015) and Shandong Provincial Hospital Affiliated to Shandong University (SPH IRB# 16-0061), sample collection began in July 2016. All participants (mothers) who visited the Institute of Child Health Care and agreed to serve as fecal donors provided written informed consent and questionnaire data sheets, in accordance with national legislation and the Code of Ethical Principles for Medical Research Involving Human Subjects of the World Medical Association (Declaration of Helsinki). The specimen bank for the Children’s Microbiome Initiative at Qilu Children’s Hospital, in collaboration with the Shandong Provincial Hospital, has collected over 2000 samples from individuals with various diseases and healthy individuals. Parental samples were obtained whenever possible. From this specimen bank, we used stool samples from 59 mother–child pairs of ASD children and 30 matched mother–child pairs of healthy (neurotypical) children for the current gut microbiome study.

**Supplementary Figure S5:**
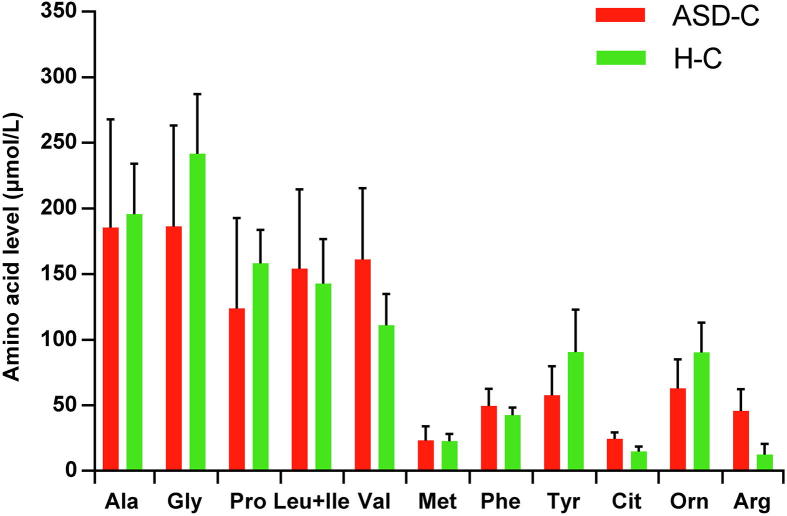
**Amino acid level in blood of ASD-C and H-C subjects**

ASD patients with clinically significant inflammatory symptoms were excluded. We also collected the clinical index of amino acid level, including alanine, glycine, proline, leucine + isoleucine, valine, methionine, phenylalanine, tyrosine, citrulline, ornithine, Arg (Figure S5). Patients with ASD were consecutively admitted to the Institute of Child Health Care of the Qilu Children’s Hospital, and ASDs were diagnosed according to the Diagnostic and Statistical Manual of Mental Disorders, 5th Edition (DSM-5) [60], and evaluated using the Autism Diagnostic Observation Schedule and Autism Behaviour Checklist and the proposed criteria for ASD in the DSM-5 [61]. All participants in this study received a Chinese-based diet provided daily by the hospital cafeteria, and no antibiotics, probiotics, or prebiotics had been taken within 3 months before sampling. No patients were treated with anti-inflammatory or antioxidant drugs. The Institute of Child Health Care of Qilu Children’s Hospital is a center specialized for training and treatment of ASD. ASD children included in the current study were all from the same six-month training class, during which they stayed in the hospital ward, ate the hospital meal provided daily by the hospital cafeteria, and received training together with their mothers. At the same time, the Research Institute of Pediatrics of Qilu Children’s Hospital conducts another clinical study, for which healthy children and their parents (hospital employees including doctors and nurses) were surveyed by having the same meal provided by the hospital cafeteria for three months. All stools were sampled at the end of the third month.

### Sample collection, DNA extraction, and sequencing

Stool samples from enrolled patients were collected with sterilized 2-ml tubes containing pure ethanol, aliquoted, and frozen at −80 °C until DNA extraction. Total DNA extraction from fecal samples (250 mg, wet weight) was performed using a FastDNA SPIN Kit for Feces (MP Biomedicals, Santa Ana, CA, USA) according to the manufacturer’s instructions. NanoDrop ND-1000 Spectrophotometer (Nucliber) was used for DNA quantification with an equivalent of 1 μL of each sample. For each DNA sample, we amplified respectively the bacterial 16S rRNA genes using a primer set specific for V1-V2 variable region of 16S rRNA gene with the universal primers F27 (5′-AGAGTTTGATCMTGGCTCAG-3′) and R338-I (5′-GCWGCCTCCCGTAGGAGT-3′) and R338-II (5′-GCWGCCACCCGTAGGTGT-3′). Amplicons were first purified using the QIAquick PCR Purification Kit (Qiagen, Barcelona, Spain), quantified using a NanoDrop ND-1000 Spectrophotometer (Nucliber) and then mixed at the same concentration. The mixed amplicons (2 nM) were then sequenced by Illumina HiSeq sequencer (Illumina Inc., San Diego, CA, USA), as described in the standard Illumina platform protocols. In this study, all sequencing data were uploaded to the NCBI SRA database (accession number: PRJNA453894) and can be accessed at https://www.ncbi.nlm.nih.gov/sra/. All sequencing data can also be viewed at NODE (http://www.biosino.org/node) by pasting the accession No. OEP000294 into the text search box or through the URL: http://www.biosino.org/node/project/detail/OEP000294.

### Analysis of 16S rRNA gene sequences

Raw FASTQ files were processed demultiplexed, quality-filtered by Trimmomatic according to the previous description [62]. The files were then merged by FLASH with the following criteria. (a) When an average quality score <20 was obtained on a 50 bp sliding window, all readers were truncated at any site. (b) Primers were exactly matched and allowed for a 2-nucleotide mismatch. Deleted readers contained ambiguous bases. (c) Merged sequences that are longer than 10 bp in overlap based on overlapping sequences. High-throughput sequencing analysis of bacterial rRNA genes was processed using the Quantitative Insights into Microbial Ecology (QIIME, version 1.9.1) software suite [63], according to the QIIME tutorial (http://qiime.org/). Chimeric sequences were, subsequently, removed using usearch61 [64] with *de novo* models. Selected high-quality sequences were clustered against the 2013 Greengenes (13_8 release) ribosomal database (97% reference data set). Sequences that did not hit the reference sequence collection were subsequently assigned to *de novo* OTUs with UCLUST algorithm in QIIME with a similarity threshold of 97%. The taxonomic identity of each OTU was determined using the RDP Classifier [65] within QIIME and the Greengenes reference data set. Alpha and beta diversity metrics from the final OTU table without singletons were obtained within the QIIME pipeline. Principal component analysis (PCA) was complemented by hierarchical clustering using unweighted pair group method with arithmetic mean (UPGMA) clustering (also known as average linkage) on the distance matrix of OTU abundance. The QIIME package was used to obtain a Newick formatted tree.

The LEfSe was used to explore potential bacterial biomarkers associated with different groups. LEfSe is an algorithm for high-dimensional biomarker discovery, which uses LDA to estimate the effect size of each classification unit that differs between cases and controls. Besides detecting significant features, LEfSe also ranks features based on effect size, putting features that account for most of the biological difference at the top [66]. The selected biomarkers were classified and analyzed by SVM classifier of R package e1071. The performance of the predictive model was evaluated using five-fold cross-validation. The ROCR R package was used to calculate the ROC curve and the AUC value [67].

### Statistical analysis

In order to account for any bias caused by nonuniformity sequencing depth, the minimum number of sequences present in any given sample from a sample category was selected randomly before calculating community-wide dissimilarity measures (alpha diversity and beta diversity), and we rarefied the sequence data in QIIME to a sequencing depth of 11,000 per sample for both diversity analyses. Principal coordinates were computed for the unweighted distance matrices and used to generate principal coordinate analysis (PCoA) plots using evenly sampled OTU abundances. Based on the marker gene data and a database of reference genomes, the functional composition of a metagenome was predicted with PICRUSt, as described by Langille et al. [68]. Graphical representations of the results were created using STAMP [69] and the calculation of P values was performed with Kruskal–Wallis H-tests and Welch’s t-tests. The P values were corrected by False Discovery Rates (FDR) to control for multiple hypothesis testing. Benjamini–Hochberg procedure was used to control the FDR at 5%. Differences were considered statistically significant when the FDR corrected *P* value was <0.05.

## Competing interests

The authors declare that they have no competing interests.

## Authors' contributions

LZ conceived and designed the study, drafted the initial manuscript, and reviewed and revised the manuscript. ZG and GL conceptualized and designed the study, coordinated and supervised data collection, and critically commented on the important intellectual content of the manuscript. GPZ and YJ, critically reviewed and revised the manuscript. NL, DZ, YL, and LHZ, designed the clinical settings, went through ethic evaluation process, performed consents and questionnaire data sheets with patients, collected clinical samples and patients’ information, and reviewed and revised the manuscript. JJY, YW, CL, JZ, BC, CZ, JW, and GYZ designed the data collection instruments, collected data, carried out the initial analyses, and reviewed and revised the manuscript. All authors approved the submission of the final manuscript and agreed to be responsible for all aspects of the work.

## Figures and Tables

**Supplementary Figure S4:**
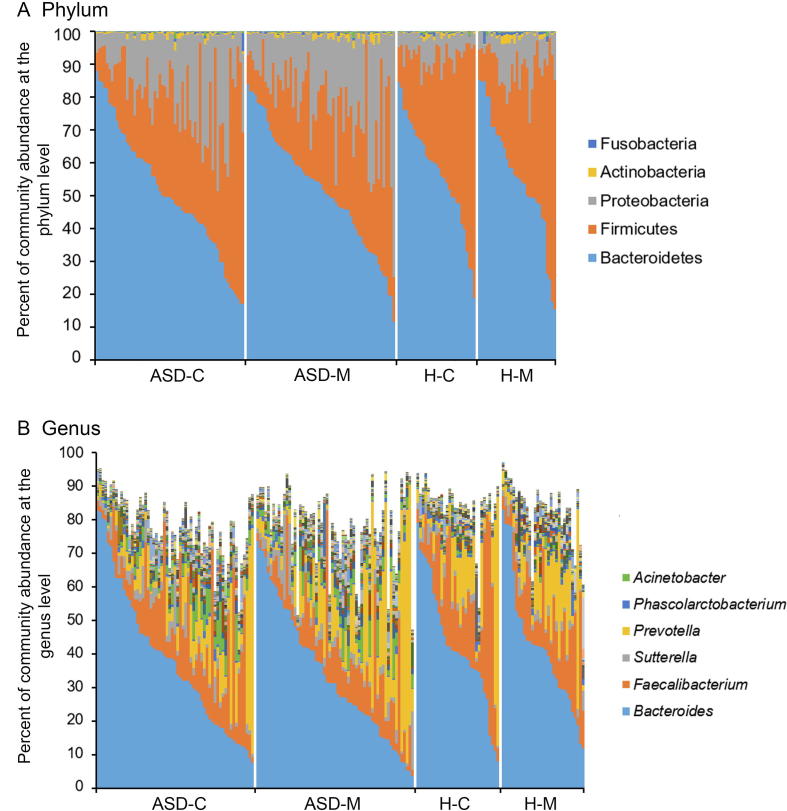
**LEfSe analysis between the autism groups and the control groups** Histogram of the LDA scores computed for OTUs differentially abundant between different groups. **A.** ASD-C *vs.* H-C. **B.** ASD-M *vs.* H-M. **C.** ASD-M+C *vs.* H-M+C. Health-enriched OTUs are indicated with positive LDA scores, and OTUs enriched in autism have negative scores. The LDA score indicates the effect size and ranking of each differentially abundant taxon. Cladograms generated by LEfSe indicate differences in the bacterial taxa between different groups. **D.** ASD-C *vs.* H-C. **E.** ASD-M *vs.* H-M. **F.** ASD-M+C *vs.* H-M+C. Nodes in red indicate taxa that were enriched in autism group compared to those in health controls, while nodes in green indicate taxa that were enriched in health controls compared to those in the autism group.
