# Supplementary material for: Correlation of Gut Microbiome Between ASD Children and Mothers and Potential Biomarkers for Risk Assessment
Source: Genomics Proteomics Bioinformatics. 2019 Apr 23;17(1):26–38. doi: 10.1016/j.gpb.2019.01.002 (PMC6520911; doi:10.1016/j.gpb.2019.01.002)
Supplement: Supplementary Table S1 [file mmc1.docx]

**Table S1 Clinical characteristics of ASD-C and H-C subjects**

| **Parameter** | **ASD-C** | **H-C** |
| --- | --- | --- |
| No. of subjects | 59 | 30 |
| Age (year), range (mean± SD) | 2–7 (4.03 ± 1.20) | 2–10 (4.93 ± 1.30) |
| Gender |  |  |
| Male (%) | 50 (84.8%) | 20 (66.6%) |
| Female (%) | 9 (15.2%) | 10 (33.3%) |
| Geographical distribution | Shandong Province, China | Shandong Province, China |
| Birth weight (kg), range (mean ± SD) | 1.5–4.8 (3.41 ± 0.62) | 2.4–6.1 (3.52 ± 0.65) |
| Birth height (cm), range (mean ± SD) | 47–75 (51.41 ± 3.81) | 48–109 (52.47 ± 9.27) |
| Current weight (kg), range (mean ± SD) | 11.5–35 (18.38 ± 5.21) | 12.5–50 (21.67 ± 6.89) |
| Current Height (cm), range (mean ± SD) | 83–138 (104.86 ± 12.48) | 88–140 (114.74 ± 8.29) |
| Mother’s reproductive age, range (mean ± SD) | 22–41 (28.36 ± 3.73) | 22–42 (27.41 ± 3.73) |
| C-section | 59.23% | ND |
| Copper (μM) | 16.86 ± 3.37 | ND |
| Zinc (μM) | 64.37 ± 10.65 | ND |
| Ferrum (mM) | 7.57 ± 1.21 | ND |
| Lead (mM) | 25.36 ± 14.73 | ND |
| Kalium (mM) | 4.49 ± 1.43 | ND |
| Natrium (mM) | 140.07 ± 43.07 | ND |
| Chlorine (mM) | 104.09 ± 34.27 | ND |
| Anion gap (mM) | 17.93 ± 10.34 | ND |
| Carbon dioxide (mM) | 14.63 ± 6.89 | ND |
| Calcium (mM) | 6.35 ± 8.48 | ND |
| Phosphorus (mM) | 1.59 ± 0.51 | ND |
| Magnesium (mM) | 0.79 ± 0.27 | ND |
| Creatine kinase isoenzyme (U/l) | 16.27 ± 7.8 | ND |
| Glutamic pyruvic transaminase (U/l) | 14.43 ± 6.27 | ND |
| Glutamic pyruvic aminotransferase (U/l) | 30.74 ± 10.69 | ND |
| Free triiodothyronine (pM) | 5.61 ± 1.06 | ND |
| Thyroid stimulating hormone (μIU/ml) | 1.78 ± 0.91 | ND |
| Free thyroxine (pM) | 15.17 ± 3.11 | ND |

*Note:* ND, not determined.
