# Supplementary material for: Correlation of Gut Microbiome Between ASD Children and Mothers and Potential Biomarkers for Risk Assessment
Source: Genomics Proteomics Bioinformatics. 2019 Apr 23;17(1):26–38. doi: 10.1016/j.gpb.2019.01.002 (PMC6520911; doi:10.1016/j.gpb.2019.01.002)
Supplement: Supplementary Table S2 [file mmc2.docx]

**Table S2 The biomarkers discovered by LEfSe analysis (LDA score > 2)**

| **Classification level** | **Increased in ASD-C** | **Increased in H-C** | **Increased in ASD-M** | **Increased in H-M** |
| --- | --- | --- | --- | --- |
| **Species** | *Stenotrophomonas* *geniculata*, *Brevundimonas diminuta*, *Acinetobacter rhizosphaerae*, and *Acinetobacter* *johnsonii* | *Melaninogenica* | *Stenotrophomonas* *geniculata*, *Pseudoxanthomonas mexicana*, *Brevundimonas diminuta*, *Acinetobacter rhizosphaerae*, and *Acinetobacter* *johnsonii* | *Prevotella* and *Melaninogenica* |
| **Genus** | *Comamonas*, *Clostridium*, *Haemophilus*, *Stenotrophomonas*, *Wautersiella*, *Pseudomonas*, *Agrobacterium*, *Sphingomonas*, *Brevundimonas*, *Enhydrobacter*, *Chryseobacterium*, *Streptococcusn*, and *Acinetobacte* | *Prevotella* | *Stenotrophomonas*, *Pseudoxanthomonans*, *Comamonas*, *Agrobacterium*, *Brevundimonas*, *Sphingomonas*, *Deinococcus*, *Pseudomonas*, *Enhydrobacter*, *Chryseobacterium*, *Streptococcus*, and *Acinetobacter* | *Bacteroides*，*Fusobacterium*，and *Porphyromonas* |
| **Family** | Aeromonadaceae, Gemellaceae, Rhodocyclaceae, Pasteurellaceae, Rhizobiaceae, Comamonadaceae, Pseudomonadaceae, Oxalobacteraceae, Sphingomonadaceae, Caulobacteraceae, Weeksellaceae, Stretococcaceae, Xanthomonadaceae, Alcaligenaceae, Enterobacteriaceae, and Moraxellaceae | Ruminococcaceae, Paraprevotellaceae | Chitinophagaceae, Rhodocyclaceae, Aeromonadaceae, Flavobacteriaceae, Gemellaceae, Sphingobacteriaceae, Rhizobiaceae, Oxalobacteraceae, Comamonadaceae, Deinococcaceae, Sphingomonadaceae, Pseudomonadaceae, Caulobacteraceae, Weeksellaceae, Xanthomonadaceae, Streptococcaceae, Moraxellaceae, and Enterobacteriacea | Bacteroidaceae and Fusobacteriaceae |
