# Supplementary material for: Correlation of Gut Microbiome Between ASD Children and Mothers and Potential Biomarkers for Risk Assessment
Source: Genomics Proteomics Bioinformatics. 2019 Apr 23;17(1):26–38. doi: 10.1016/j.gpb.2019.01.002 (PMC6520911; doi:10.1016/j.gpb.2019.01.002)
Supplement: Supplementary Table S3 [file mmc3.docx]

**Table S3**  **Taxa that differ in the fecal microbiome among ASD-C, H-C, ASD-M and H-M (results of Venn)**

| **Groups** | **H-M** | **ASD-M** | | **ASD-C** | **H-C** | **Health** |
| --- | --- | --- | --- | --- | --- | --- |
| **TAXA** |  | Anaerolineae | *Morganella morganii* |  |  |  |
|  |  | Gemm-3 | *Pseudomonas stutzeri* | VC2_1_Bac22 |  |  |
|  |  | Thermomicrobia | *Psychrobacter sanguinis* | mitochondria |  |  |
|  |  | TK10 | *Rhodococcus* | Promicromonosporaceae |  |  |
|  |  | Beijerinckiaceae | *Spirosoma* | *Achromobacter* |  |  |
|  | *Butyrivibrio* | C111 | *Streptococcus luteciae* | *Adlercreutzia* |  |  |
|  | Paenibacillaceae | Nocardiaceae | *Streptococcus sobrinus* | *Anaeroplasma* | Elusimicrobiaceae | *Bacteroides ovatus* |
|  | *Dyadobacter* | Pseudanabaenaceae | *Truepera* | *Corynebacterium variabile* | ML615J-28 | *Abiotrophia* |
|  | *CF231* | *Akkermansia muciniphila* | *Zoogloea* | *Epulopiscium* |  |  |
|  | *Mycoplasma* | *Ardenscatena* | B07_WMSP1 | *Hydrogenophilus* |  |  |
|  |  | *Cobetia* | iii1-15 | *Mesorhizobium* |  |  |
|  |  | *Dechloromonas* | JG30-KF-CM45 | *Salinicoccus* |  |  |
|  |  | *Friedmanniella* | MWH-UniP1 | *Sphingobium xenophagum* |  |  |
|  |  | *Lactobacillus ruminis* | Myxococcales | Solirubrobacterales |  |  |
|  |  | *Lactococcus* |  |  |  |  |
