# Supplementary material for: Correlation of Gut Microbiome Between ASD Children and Mothers and Potential Biomarkers for Risk Assessment
Source: Genomics Proteomics Bioinformatics. 2019 Apr 23;17(1):26–38. doi: 10.1016/j.gpb.2019.01.002 (PMC6520911; doi:10.1016/j.gpb.2019.01.002)
Supplement: Supplementary Table S4 [file mmc4.docx]

**Table S4 Evaluation of all biomarker combinations (in accordance with Figure 3B and 3C)**

|  | **ASD-C *vs*. H-C** | | | | **ASD-M *vs*. H-M** | | | |
| --- | --- | --- | --- | --- | --- | --- | --- | --- |
|  | ***P* value** | **95% CI** | **Sensitivity** | **Specificity** | ***P* value** | **95% CI** | **Sensitivity** | **Specificity** |
| **Fold 1** | 0.000162 | 64-100 | 100 | 100 | 0.003555 | 72-100 | 66.66667 | 63.63636 |
| **Fold 2** | 1.98E-05 | 65-100 | 100 | 100 | 0.000595 | 80-100 | 42.85714 | 91.66667 |
| **Fold 3** | 0.000833 | 94-100 | 83.33333 | 91.66667 | 0.001023 | 79-100 | 33.33333 | 91.66667 |
| **Fold 4** | 0.000215 | 84-100 | 83.33333 | 91.66667 | 0.000108 | 94-100 | 100 | 100 |
| **Fold 5** | 0.003448 | 70-100 | 50 | 75 | 0.000108 | 94-100 | 100 | 100 |
