# Supplementary material for: Correlation of Gut Microbiome Between ASD Children and Mothers and Potential Biomarkers for Risk Assessment
Source: Genomics Proteomics Bioinformatics. 2019 Apr 23;17(1):26–38. doi: 10.1016/j.gpb.2019.01.002 (PMC6520911; doi:10.1016/j.gpb.2019.01.002)
Supplement: Supplementary Table S5 [file mmc5.docx]

**Table S5 Effect of age, gender, and history of GI problem on the discovered biomarkers for ASD-C *vs.* H-C**

| **Biomarker** | **ASD-C** | | | **H-C** | | |
| --- | --- | --- | --- | --- | --- | --- |
|  | **Age** | **Gender** | **GI**  **problem** | **Age** | **Gender** | **GI**  **problem** |
| Betaproteobacteria | 0.479577 | 0.774929 | 0.6093034 | 0.656652 | 0.577958 | 0.632939 |
| Burkholderiales | 0.378924 | 0.528275 | 0.6917461 | 0.668042 | 0.71566 | 0.634437 |
| Pseudomonadales | 0.26848 | 0.335158 | 0.7130145 | 0.617163 | 0.439729 | 0.34578 |
| Moraxellaceae | 0.236807 | 0.335699 | 0.7659946 | 0.721364 | 0.418303 | 0.912851 |
| *Acinetobacter* | 0.307742 | 0.249773 | 0.7577924 | 0.736645 | 0.955403 | 0.531116 |

*Note*: The effects of each of the two factors, *i.e.*, age and gender on the validated biomarkers were examined within each of the 2 clinical categories by the R built-in one way ANOVA test. The effect was considered significant if *P* < 0.05.
