# Supplementary material for: Correlation of Gut Microbiome Between ASD Children and Mothers and Potential Biomarkers for Risk Assessment
Source: Genomics Proteomics Bioinformatics. 2019 Apr 23;17(1):26–38. doi: 10.1016/j.gpb.2019.01.002 (PMC6520911; doi:10.1016/j.gpb.2019.01.002)
Supplement: Supplementary Table S6 [file mmc6.docx]

**Table S6 Effect of Age and history of GI problem on the discovered biomarkers for ASD-M *vs.* H-M**

| **Biomarker** | **ASD-M** | | **H-M** | |
| --- | --- | --- | --- | --- |
|  | **Age** | **GI problem** | **Age** | **GI problem** |
| Flavobacteriia | 0.8506098 | 0.605908 | 0.9473266 | 0.716337 |
| Gammaproteobacteria | 0.6510482 | 0.083727 | 0.9466317 | 0.368244 |
| Flavobacteriales | 0.8506098 | 0.605908 | 0.9473266 | 0.716336 |
| Weeksellaceae | 0.8470501 | 0.610121 | 0.9474405 | 0.669912 |
| Enterobacteriaceae | 0.8826499 | 0.222143 | 0.98178 | 0.373652 |
| Enterobacteriales | 0.8826499 | 0.222143 | 0.98178 | 0.373656 |

*Note*: The effects of each of the two factors, *i.e.*, age and gender on the validated biomarkers were examined within each of the 2 clinical categories by the R built-in one way ANOVA test. The effect was considered significant if *P* < 0.05.
