# Supplementary material for: Correlation of Gut Microbiome Between ASD Children and Mothers and Potential Biomarkers for Risk Assessment
Source: Genomics Proteomics Bioinformatics. 2019 Apr 23;17(1):26–38. doi: 10.1016/j.gpb.2019.01.002 (PMC6520911; doi:10.1016/j.gpb.2019.01.002)
Supplement: Supplementary Table S7 [file mmc7.docx]

**Table S7 A summary of all previous microbiome studies on ASD children**

| **Study group** | **Methodology** | **Significantly higher in ASD** | **Significantly lower in ASD** | **Ref.** |
| --- | --- | --- | --- | --- |
| 10 ASD, 10 PDD-NOS, 10 CON | Pyrosequencing and bacterial culture, feces | *Caloramator*, *Sarcina*, and *Clostridium*;  Bacteroidetes (ASD and PDD-NOS) | *Bifidobacterium* spp., Eubacteriaceae | [28] |
| 13 ASD, 8 CON | Bacterial culture, feces | *Clostridium* and *Ruminococcus* spp. |  | [37] |
| 58 ASD, 12 SIB, 10 CON | Fluorescent *in situ* hybridization, feces | *Clostridium histolyticum* group (*Clostridium* clusters I and II) |  | [33] |
| 15 ASD with GI symptoms, 7 CON with GI symptoms | Pyrosequencing and quantitative PCR, ileal and cecal biopsies | Cumulative level of Firmicutes, Proteobacteria; *Sutterella* spp. | Bacteroidetes | [29] |
| 21 ASD, 19 CON | Pyrosequencing, duodenal | *Burkholderia*, *Clostridium* | *Neisseria*, *Bacteroides*, *Escherichia coli* | [24] |
| 10 ASD, 9 SIB, 10 CON | Real-time PCR, feces | *Desulfovibrio* spp., *Desulfovibrio* spp. | *Lactobacillus* spp. | [25] |
| 20 ASD, 20 CON | Pyrosequencing, feces |  | Genus level: *Prevotella, Coprococcus*  family level: unclassified Veillonellaceae | [27] |
| 58 ASD, 39 CON | Bacterial culture, feces | *Lactobacillus* spp., *Bacillus* spp. | *Bifidobacterium* spp., *Enterococcus* spp.,  *Klebsiella oxytoca* | [31] |
| 33 ASD, 7 SIB, 8 CON | Pyrosequencing, feces | *Desulfovibrio*, *Bacteroides vulgatus*; Bacteroidetes | Firmicutes | [32] |
| 15 ASD, 8 CON | Quantitative PCR, feces | *Clostridium* clusters I and XI, *Clostridium bolteae* |  | PMID: 15528506 |
| 23 ASD, 22 SIB, 9 CON | Quantitative PCR, feces | *Bacteroides fragilis* in ASD subjects with GI symptoms only  (9 of 23) | *Akkermansia muciniphila* (ASD and SIB); *Bifidobacterium* spp.  (ASD only) | PMID: 21784919 |

*Note*: ASD, autism spectrum disorder; PDD-NOS, pervasive developmental disorder not otherwise specified;  CON, control subject; SIB, non-autistic sibling; GI, gastrointestinal.
