# Supplementary material for: Correlation of Gut Microbiome Between ASD Children and Mothers and Potential Biomarkers for Risk Assessment
Source: Genomics Proteomics Bioinformatics. 2019 Apr 23;17(1):26–38. doi: 10.1016/j.gpb.2019.01.002 (PMC6520911; doi:10.1016/j.gpb.2019.01.002)
Supplement: Supplementary Table S8 [file mmc8.docx]

**Table S8 Similar results with previous studies**

| **Species** | **Cavity** | **Species population size compared with normal (if commensal)** | **Ref.** |
| --- | --- | --- | --- |
| β-Proteobacteria | Gut | Increased | [30] |
| Alcaligenaceae | Gut | Increased | [30] |
| *Prevotella* | Gut | Decreased | [54] |
| *Clostridium* | Gut | Increased | [32] |
